# Study of the Equivalent Stiffness of a Non-Contact Piezoelectric Actuator’s Micro-Displacement Amplification Mechanism

**DOI:** 10.3390/mi16090974

**Published:** 2025-08-25

**Authors:** Huaiyong Li, Dongya Zhang, Yusheng Lin, Yue Yang, Zhiwei Shi, Chong Li

**Affiliations:** 1 Faculty of Mechanical and Material Engineering, Huaiyin Institute of Technology, Huai’an 223003, China; zdy1600704032@163.com (D.Z.); 13951132211@163.com (Y.L.); 18360890867@163.com (Y.Y.); s312039303@163.com (Z.S.); 2 Jiangsu Key Laboratory of Advanced Manufacturing Technology, Huaiyin Institute of Technology, Huai’an 223003, China; lichong@just.edu.cn; 3 School of Mechanical Engineering, Jiangsu University of Science and Technology, Zhenjiang 212100, China

**Keywords:** non-contact piezoelectric actuator, micro-displacement amplification mechanism, filleted straight-beam flexure hinge, equivalent stiffness

## Abstract

To address the issues of mechanical wear and limited service life in conventional contact piezoelectric actuators, this study proposes a non-contact piezoelectric actuator employing compressed air for energy transmission; we elucidate its structure and operating principle. The working performance of the actuator is significantly affected by the amplification performance of its micro-displacement amplification mechanism, which itself is closely dependent on the mechanism’s stiffness. Mathematical models for both the filleted straight-beam flexure hinge and the micro-displacement amplification mechanism are established. Analytical equations for calculating the equivalent stiffness of the hinge and the mechanism are derived. The variations in the hinge’s bending stiffness and tensile stiffness, as well as the mechanism’s equivalent stiffness with key structural parameters, are investigated. The stress distribution of the micro-displacement amplification mechanism is analyzed to evaluate the rationality and reliability of its structural design. A prototype is fabricated and equivalent stiffness tests are conducted. The theoretical calculation is basically consistent with the experimental results, verifying the accuracy of the stiffness model. The results show that flexure hinge tensile stiffness significantly exceeds the bending stiffness, permitting the simplification of the hinge stiffness model. Hinge minimum thickness and beam length critically affect mechanism stiffness; reducing thickness or increasing beam length lowers stiffness, boosting displacement amplification.

## 1. Introduction

The rapid advancement of technology and the emergence of new application demands have given rise to various micromotors that are distinct from traditional electromagnetic motors [1,2,3,4,5]; among them, piezoelectric motors stand out as a successfully developed example. Piezoelectric actuators do not require magnets or coils; instead, they convert electrical energy into mechanical energy, delivering motion by exploiting the inverse piezoelectric effect of piezoelectric materials [6,7,8,9,10].

Piezoelectric actuators have been successfully applied in fields such as aerospace, microbots, biomedical science, optics, and more, due to their characteristics: compact structure, small size, lightweight, stable operations with low velocity, high torque, fast response with high displacement resolution, self-locking upon power-off, no magnetic disturbance, and easy control, etc. [11,12,13,14,15,16,17,18,19,20].

Piezoelectric actuators are categorized into contact and non-contact types based on the contact conditions between the stator and the mover. The research on contact-type actuators have been widely reported and the technology is relatively mature. Conversely, significant efforts focus on developing driving methods and design theories for non-contact actuators to overcome the limited service life of contact types caused by the stator-mover frictional losses [21,22,23,24,25,26].

In 1990s, Yamayoshi et al. [27] investigated a non-contact ultrasonic motor in which the fluid between the stator and the rotor was used to transmit torque, and a speed of 3000 r/m was obtained under the excitation voltage of 100 V. Hu et al. [28] developed a noncontact ultrasonic motor using liquid as the transmission medium, carried out theoretical and experimental studies, and established design guidelines for optimal motor performance. Isobe et al. [29] proposed a non-contact ultrasonic motor that enables micro-positioning of the rotor with a minimum step width of 0.15 μm, achieving contact-free and precise positioning capabilities. Stepanenko et al. [30] developed a standing wave-based non-contact rotary ultrasonic motor featuring an annular stator that drives a blade-shaped rotor through in-plane bending vibrations. The rotational speed and direction of the motor can be precisely controlled by adjusting the excitation frequency of the stator. Gabai et al. [31] studied a non-contact ultrasonic motor based on acoustic levitation and traveling wave control. The stator of this motor is composed of three Langevin transducers, each operating at a natural frequency of 28 kHz. By independently adjusting the amplitude and phase of each transducer, different ratios of traveling and standing waves are achieved, thus enabling the modulation of the rotational torque. Shi et al. [32] developed a non-contact ultrasonic motor based on near-field acoustic levitation. Featuring a compliant structure with self-adaptive capability, the motor generates controllable tangential force and driving torque on the rotor through acoustic radiation effects. Shi et al. [33] investigated a dual-function non-contact ultrasonic motor capable of simultaneous levitation and propulsion. The motor employs a Langevin transducer with conical tip design as the stator, which generates both radial and axial acoustic levitation forces from a single vibration source, achieving integrated rotor suspension and rotation. Under a driving voltage of 200 V, the motor attained a rotational speed of 120 rpm. Chen et al. [34,35] developed a non-contact ultrasonic motor based on acoustic streaming effects, where near-field acoustic levitation generated by ultrasonic vibrations provides contactless rotor support, while the circumferential acoustic pressure gradient in the stator–rotor air gap produces driving torque. The motor achieves an output rotational speed of 28 rpm under an excitation voltage of 1430 V.

Qiu et al. [36] investigated a non-contact rotating piezoelectric motor modulated by Giant Electrorheological (GER) fluid. Compared to motors utilizing conventional electrorheological fluids, the GER-fluid-modulated motor demonstrates significantly enhanced performance. The excitation frequency of the motor was 118 Hz, and the maximum torque and rotational speed reached 1.04 mN·m and 6.98 rad·s^−1^, respectively. Xing et al. [37] investigated a non-contact piezoelectric motor modulated by an electromagnetic field, where the reciprocating motion of the piezoelectric actuator is regulated to drive the rotor in a stepwise rotation. With a driving voltage of 150 V, frequency of 3 Hz, and modulation voltage of 7 V, the motor delivers a rotational speed of 0.038 rad·s^−1^, step angle of 1°, and output torque of 6 mN·m. Wang et al. [38,39,40] studied a novel electromagnetically modulated non-contact rotary piezoelectric actuator and developed its nonlinear coupled dynamic model. Theoretical derivation and numerical analysis were conducted to fully characterize the system’s dynamic behaviors, including coupled natural frequencies, mode shapes, bifurcation phenomena, and chaotic vibrations, with quantitative criteria for chaotic vibration initiation established.

The existing non-contact piezoelectric motors are mainly based on the mechanism of ultrasonic drive or electromagnetic modulation, which can significantly improve the speed by eliminating contact friction between the stator and rotor. The ultrasonic-driven non-contact piezoelectric motors have many excellent performance features, such as simple structure, compact size, and easy control. However, they require high precision in manufacturing and assembly and have small output torque. Conversely, the electromagnetic modulation non-contact piezoelectric motors have large output torques, but they have complex structures, large size, difficulties in miniaturization, and are challenging to control.

To simultaneously achieve large output torque, a simple structure, small size, easy controls, and no contact friction, a non-contact piezoelectric actuator with compressed air as the energy transmission medium is proposed. The actuator adopts a micro-displacement amplification mechanism to amplify the micro-deformation generated by the piezoelectric stack, compresses the gas within a sealed chamber to generate high pressure, and thereby drives the rotor to rotate, achieving non-contact power transmission.

The performance (amplification ratio) of the micro-displacement amplification mechanism is critical to the actuator, as it directly determines the compression level within the sealed chamber and the resultant gas pressure required to drive the rotor. This mechanism relies on the elastic deformation of flexure hinges to transmit force and displacement; thus, these flexible hinges are its weakest part. The deformation degree of the hinges directly affects the mechanism’s amplification performance, and this deformation is closely related to the hinges’ stiffness. Therefore, in-depth research on the stiffness characteristics of the micro-displacement amplification mechanism is essential for enhancing the overall mechanical performance of the non-contact piezoelectric actuator.

In this study, the structure of the non-contact piezoelectric actuator is determined and its operating principle is illustrated. Mathematical models for the filleted straight-beam flexure hinge within the micro-displacement amplification mechanism and for the overall mechanism are established. The stiffness calculation equations of flexible hinges and amplification mechanisms are deduced. The bending stiffness, tensile stiffness of the flexible hinge, and the equivalent stiffness of the micro-displacement amplification mechanism are thoroughly explored in terms of the changes, along with the flexure hinge’s structural parameters. Analysis of the stress distribution in the micro-displacement amplification mechanism confirmed the rationality and reliability of its structural design. Furthermore, the prototype is fabricated and the equivalent stiffness tests are conducted. The theoretical calculation is basically consistent with the experimental results, verifying the accuracy of the stiffness model. The results demonstrate that: (1) the tensile stiffness of the flexure hinge is significantly higher than its bending stiffness, allowing the hinge stiffness in the micro-displacement amplification mechanism to be simplified as bending stiffness for modeling purposes; (2) the minimum thickness and straight-beam length of the hinge significantly influence the mechanism’s equivalent stiffness; (3) reducing the minimum thickness or increasing the straight-beam length effectively reduces the equivalent stiffness, thereby enhancing the displacement amplification capability. These findings provide a theoretical foundation for further study about structure, dynamic performance and control strategies of non-contact piezoelectric actuators.

## 2. Structure and Operating Principle

**Figure 1:**
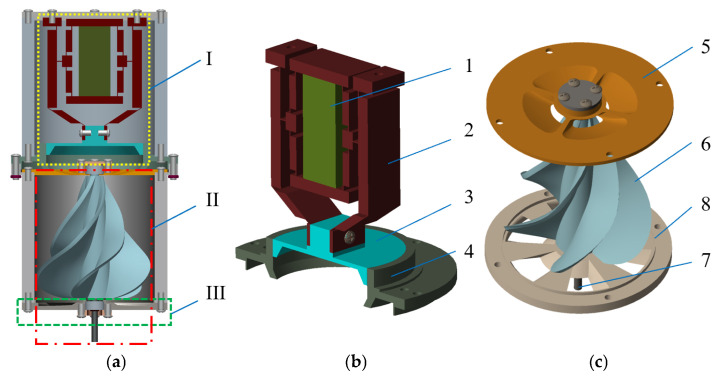
Structure of the non-contact piezoelectric actuator. (**a**) Overall structure; (**b**) driving component; (**c**) transmission component; I—driving component; II—transmission component; III—base component; 1—piezoelectric stack; 2—displacement amplification mechanism; 3—piston; 4—piston sleeve; 5—upper bearing seat; 6—gyroscopic rotor; 7—output shaft; 8—lower bearing seat.

Figure 1 shows the non-contact piezoelectric actuator, comprising a driving component, a transmission component, and a base component. The driving component consists of a piezo-electric stack, a displacement amplification mechanism, a piston, and a piston sleeve. The transmission component is composed of an upper bearing seat, a gyroscopic rotor, an output shaft, and a lower bearing seat. Upon assembly, the piston and piston sleeve from the driving component, together with the upper bearing seat from the transmission component, form a sealed pressure chamber with a variable volume. As the piezoelectric stack exhibits limited displacement output under voltage excitation, a displacement amplification mechanism is integrated to amplify its stroke, thereby enabling effective axial motion of the piston along the sleeve.

**Figure 2:**
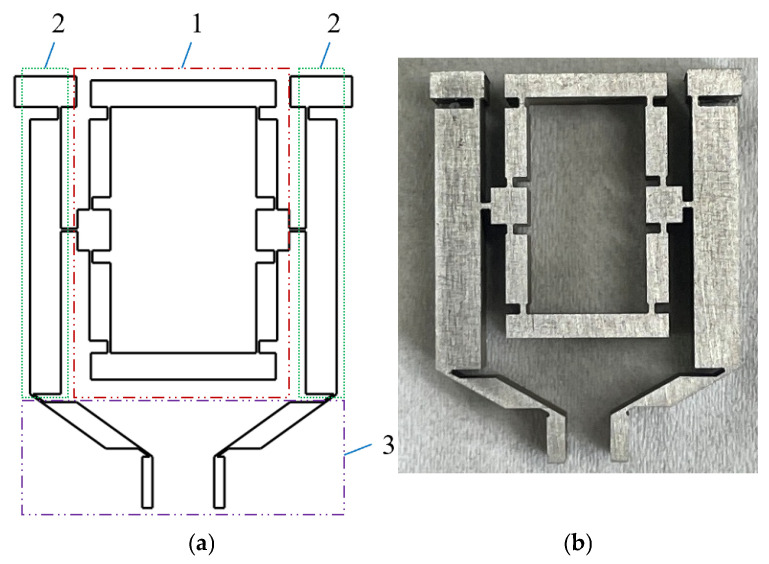
Structure of the micro-displacement amplification mechanism. (**a**) Structural components; (**b**) physical prototype; 1—bridge structure I; 2—lever structure; 3—bridge structure II.

This micro-displacement amplification mechanism is a symmetric three-stage serial structure based on a filleted straight-beam flexure hinge, which is composed of bridge structure I, a lever structure, and bridge structure II (see Figure 2). Its operational principle relies on elastic micro-angular deformation induced within the flexure hinges’ thin-walled zones to facilitate relative rotation between rigid links. This design integrates the characteristics of flexible mechanisms and has comprehensive advantages such as a compact structure, light weight, no friction pairs (no assembly/lubrication required), high motion sensitivity and rapid dynamic response.

**Figure 3:**
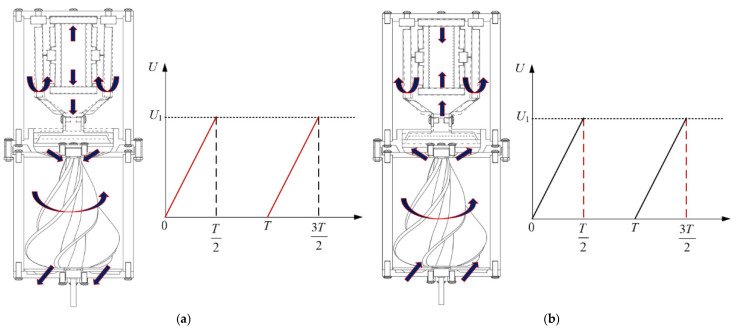
Operational principle of the non-contact piezoelectric actuator. (**a**) The motion of the driving component after the actuator has applied a rising saw-tooth wave voltage signal (the red solid lines); the black solid line denotes the maximum displacement position reached by each component of the actuator under a rising saw-tooth wave voltage signal. The black dashed line represents the initial operational position, which is also the position returned to by each component after the application of the descending saw-tooth wave voltage signal in the first half of the cycle. (**b**) the motion of the driving component after the actuator has applied a descending saw-tooth wave voltage signal (the red dashed lines). The black dashed line corresponds to the black solid line in (**a**), both representing the maximum displacement position. Conversely, the black solid line in Figure (**b**) indicates the initial operational position returned to by each component under a descending saw-tooth wave voltage signal. Note: the arrows in Figure 3 indicate the direction of structural movement at specific locations within the work area.

Figure 3 shows the operation principle of the non-contact piezoelectric actuator. The actuator is powered by a piezoelectric stack embedded in a displacement amplification mechanism. When a rising saw-tooth wave voltage signal is applied, the piezoelectric stack generates a minute displacement along its height direction due to the inverse piezoelectric effect. This micro-displacement is transmitted to bridge structure I, resulting in amplified inward contraction displacements at its lateral (left and right) sides. These contractions drive the lever structures, which are serially connected to the lateral sides of bridge structure I and pivotally fixed at their bases, to undergo inward counter-rotation relative to each other. This rotation subsequently drives bridge structure II, which is serially connected to the ends of the two lever structures, to undergo synchronized inward contraction motion. As the opposite end of bridge structure II is rigidly fixed to the piston and thus cannot contract further inward, it generates an amplified downward displacement along the longitudinal direction. This displacement drives the piston to move downward along the inner cavity of the piston sleeve. Consequently, the volume of the sealed cavity enclosed by the piston, piston sleeve, and upper bearing seat is reduced, compressing the air within it and increasing its pressure. The compressed air is then ejected through the circular truncated-cone orifices located on the upper bearing seat, impinging onto the top of the rotor. This process drives rotation of the rotor, achieving non-contact power transmission.

When the electrical signal becomes a descending saw-tooth wave voltage signal, the piezoelectric stack rapidly contracts and returns to its initial state. Concurrently, the displacement amplification mechanism resets to its initial position under the restoring force of the deformed flexure hinges. This action drives the piston upwards along the inner cavity of the piston sleeve. Hence, the volume of the sealed cavity enclosed by the piston, piston sleeve, and upper bearing seat expands, creating a partial vacuum within the cavity. Due to atmospheric pressure, ambient air is drawn into the cavity through the circular truncated-cone orifices located on the upper bearing seat. This inflow restores the internal pressure to its initial state, preparing the system for the next operating cycle.

## 3. Stiffness Equation Derivation for Filleted Straight-Beam Flexure Hinge

The flexure hinge within the displacement amplification mechanism adopts a filleted straight-beam hybrid configuration, incorporating the structural features of both circular and straight-beam hinges. While retaining the wide rotational range characteristic of conventional straight-beam flexure hinges, this design effectively suppresses stress concentrations through filleted corners, thus significantly extending fatigue life.

### 3.1. Derivation of the Bending Stiffness Equation

The structural parameters of the filleted straight-beam flexure hinge include the straight-beam length a, width b, minimum thickness t, maximum thickness H, and fillet radius R (see Figure 3). According to the operational principle of the displacement amplification mechanism, limited deformation occurs during actuation, primarily concentrated within the straight-beam segment and filleted regions.

**Figure 4:**
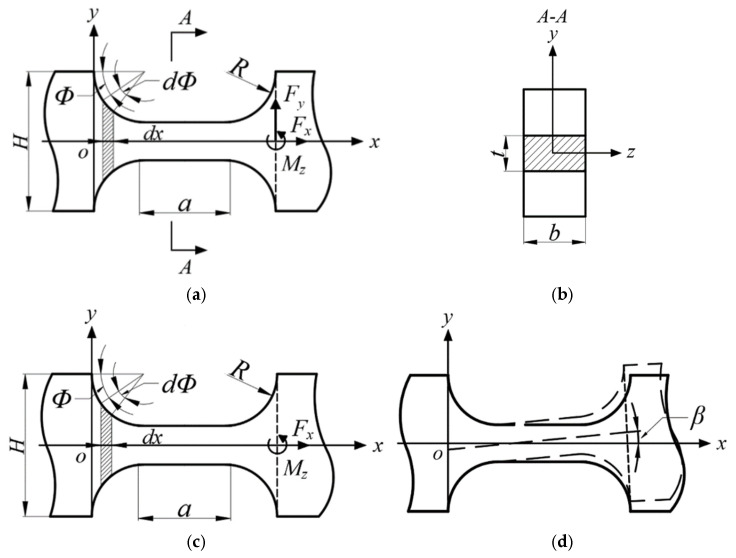
The filleted straight-beam flexure hinge. (**a**) Structural parameters and forces; (**b**) cross-sectional structure; (**c**) structural parameters and equivalent forces; (**d**) bending deformation.

Based on the small deformation theory of the cantilever beam, it is assumed that the force of the filleted straight-beam flexure hinge is as shown in Figure 4a. In its planar mechanical model, one end of the flexible hinge is fixed, and the longitudinal load of the vertical neutral surface produces a torque relative to the fixed end of the hinge, which is consistent with the torque around the center of rotation of the hinge in terms of kinematic characteristics. To simplify the analysis, the rotational torque generated by the load relative to the fixed end along the longitudinal axis of the flexible hinge and the torque around the rotation center of the hinge can be uniformly expressed as the torque around the rotation center of the hinge. Therefore, the other end of the flexible hinge is subjected to a torque M*_z_* that causes the hinge to undergo bending deformation, and a force *F_x_* that causes a slight linear displacement of the hinge along the x-axis, as shown in Figure 4c.

As shown in Figure 4d, the torque M*_z_* drives the filleted straight-beam flexure hinge to generate a micro-angular displacement *β* around the *z*-axis. This deformation fundamentally arises from the integration of bending deformations over infinitesimal rectangular cross-section segments d*x* along the beam length. The coordinate system is established as follows: the axis of the neutral layer of the beam before deformation is the *x*-axis, the normal direction of the neutral layer (vertical upward) is the *y*-axis, the *z*-axis follows the rules of the right-hand Cartesian coordinate system, and the *xy*-plane is the longitudinal symmetry plane of the beam. After deformation, the beam axis forms a continuous curve within the *xy*-plane, and its deflection function is denoted as *y* = f(*x*). According to small-deflection bending theory, given that the actual angular displacement *β* is very small, it satisfies:

(1) $$\beta \approx \tan\beta =\frac{dy}{dx}$$

According to the theory of mechanics of materials, under pure bending conditions, the bending moment M relates to the beam axis curvature κ through the constitutive equation:

(2) $$\kappa =\frac{M_{z}}{EI(x)}=\frac{\frac{d^{2}y}{dx^{2}}}{{\left[1+{\left(\frac{dy}{dx}\right)}^{2}\right]}^{\frac{3}{2}}}$$

where κ denotes the curvature of the deflection curve, E is Young’s modulus of the material, M*_z_* represents the bending moment about the *z*-axis, and *I*(*x*) represents the second moment of area of the cross-section.

Then, *I*(*x*) is given by:

(3) $$I(x)=\frac{bh^{3}(x)}{12}$$

where *h*(*x*) defines the cross-sectional height of the infinitesimal beam segment, and b is the hinge’s width.

In the kinematic analysis of flexure hinges, for straight-beam segments, the constant cross-sectional height is *h*(*x*) = t, but, for circular arc segments, the height varies as *h*(*x*) = 2R + t − 2Rsin*φ*, where *φ* is the angular coordinate, R is the fillet radius, and t denotes the minimum thickness of the hinge. The differential arc length follows d*x* = Rd*φ*, which can be approximated as d*x* ≈ Rsin*φ*d*φ* when *φ* is sufficiently small.

Given the operational characteristics of the non-contact piezoelectric actuators, the bending deflection *y* in the flexure hinges of the micro-displacement amplification mechanism is much smaller than the total hinge length a + 2R. Based on the small-deflection assumption (rotational angles satisfy *θ* ≪ 1), the term (d*y*/d*x*)^2^ in Equation (2) can be ignored. Consequently, it can be simplified as:

(4) $$\frac{M_{z}}{EI(x)}=\frac{d^{2}y}{dx^{2}}$$

Substituting Equation (4) into Equation (1) yields:

(5) $$\beta =\frac{dy}{dx}=\int_{0}^{x}\frac{d^{2}y}{dx^{2}}dx=\int_{0}^{x}\frac{M_{z}}{EI(x)}dx$$

Thus, the bending stiffness *k*_1_ of the filleted straight-beam flexure hinge is given by:

(6) $$k_{1}=\frac{M_{z}}{\beta }=\frac{1}{\int_{0}^{\pi }\frac{12R\sin\Phi }{Eb{\left(2R+t-2R\sin\Phi \right)}^{3}}d\Phi +\int_{0}^{a}\frac{12}{Ebt^{3}}dx}=\frac{1}{\frac{12u_{1}}{EbR^{2}}+\frac{12a}{Ebt^{3}}}$$

where:

$$\begin{array}{l} u_{1}=\int_{0}^{\pi }\frac{\sin\Phi }{{\left[2+\left(t/R\right)-2\sin\Phi \right]}^{3}}d\Phi =\frac{2c^{3}\left(6c^{2}+4c+1\right)}{{\left(4c+1\right)}^{2}\left(2c+1\right)}+\frac{12c^{4}\left(2c+1\right)}{{\left(4c+1\right)}^{5/2}}\arctan\sqrt{4c+1}\\ c=R/t \end{array}$$

### 3.2. Derivation of the Tensile Stiffness Equation

As shown in Figure 4, the axial load *F_x_* induces an elastic displacement *δ*a along the *x*-axis in the flexure hinge. Based on the axial deformation theory of rods in mechanics of materials, the displacement increment of an infinitesimal segment d*x* can be expressed as:

(7) $$\delta a=\frac{F_{x}dx}{EA(x)}$$

where *A*(*x*) is the cross-sectional area function varying along the hinge axis. For straight-beam segments *A*(*x*) = bt, but, for circular arc segments, *A*(*x*) = b*h*(*x*) = b(2R + t − 2Rsin*φ*); b and t are as defined above. E denotes the Young’s modulus of the material, and *F_x_* represents the axial load applied in the *x*-direction.

Therefore, the total displacement of the flexure hinge is given by:

(8) $$\Delta a=\int_{0}^{\pi }\frac{F_{x}R\sin\Phi d\Phi }{Eb\left(2R+t-2R\sin\Phi \right)}+\int_{0}^{a}\frac{F_{x}}{Ebt}dx=\int_{0}^{\pi }\frac{F_{x}\sin\Phi d\Phi }{Eb\left(2+\left(t/R\right)-2\sin\Phi \right)}+\frac{F_{x}a}{Ebt}=\frac{F_{x}u_{2}}{Eb}+\frac{F_{x}a}{Ebt}$$

where

$$u_{2}=\int_{0}^{\pi }\frac{\sin\Phi }{2+\left(t/R\right)-2\sin\Phi }d\Phi =\frac{2\left(2c+1\right)}{\sqrt{4c+1}}\arctan\sqrt{4c+1}-\frac{\pi }{2}$$

Based on the above analysis, the axial stiffness (i.e., tensile stiffness) *k*_2_ of the filleted straight-beam flexure hinge can be expressed as:

(9) $$k_{2}=\frac{F_{x}}{\Delta a}=\frac{1}{\int_{0}^{\pi }\frac{\sin\Phi d\Phi }{Eb\left[2+\left(t/R\right)-2\sin\Phi \right]}+\frac{a}{Ebt}}=\frac{1}{\frac{u_{2}}{Eb}+\frac{a}{Ebt}}$$

## 4. Numerical Example Analysis

**Figure 5:**
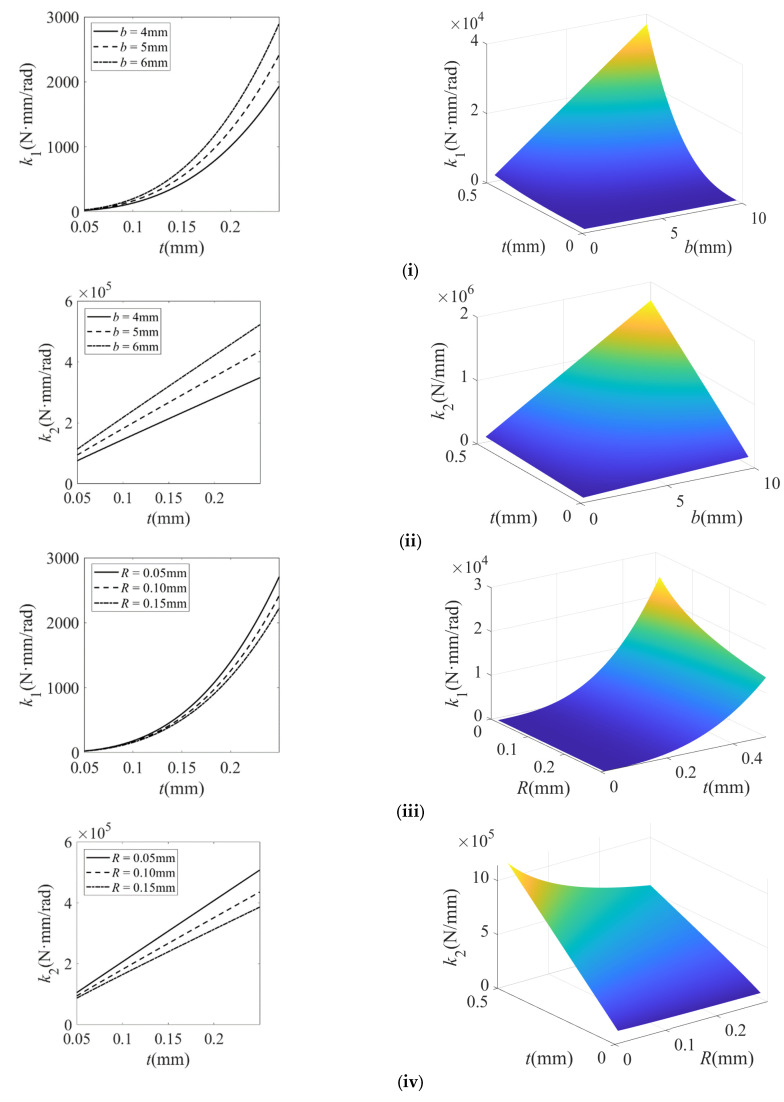

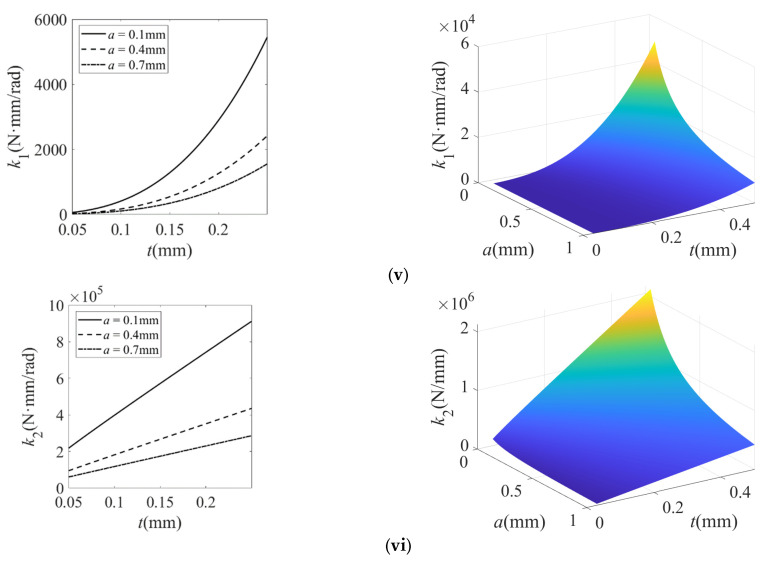
Stiffness characteristics of the filleted straight-beam flexure hinge with respect to thickness t, width b, radius R, and length a. (**i**) Bending stiffness *k*_1_ varies with b and t; (**ii**) tensile stiffness *k*_2_ varies with b and t; (**iii**) bending stiffness *k*_1_ varies with R and t; (**iv**) tensile stiffness *k*_2_ varies with R and t; (**v**) bending stiffness *k*_1_ varies with a and t; (**vi**) tensile stiffness *k*_2_ varies with a and t. In the 3D plot, the color gradient from dark blue, light blue, and light green to light yellow corresponds to the progressive increase in the stiffness of the flexible hinge.

**Figure 6:**
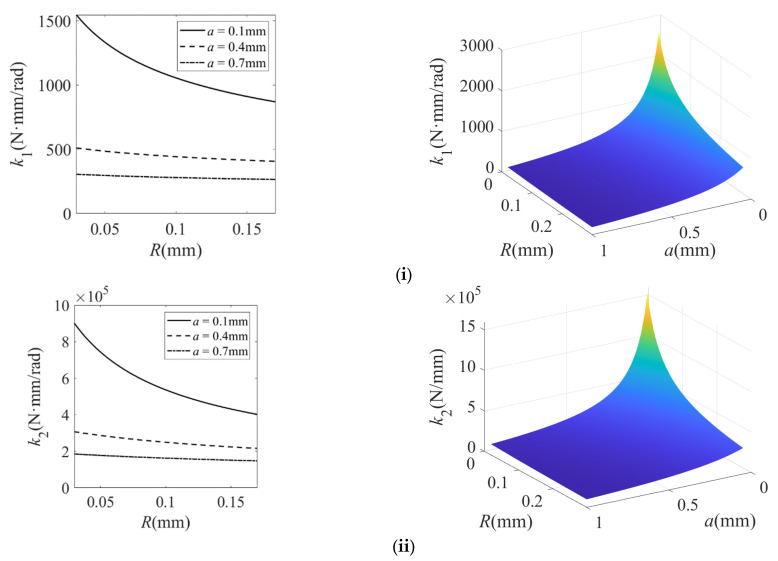

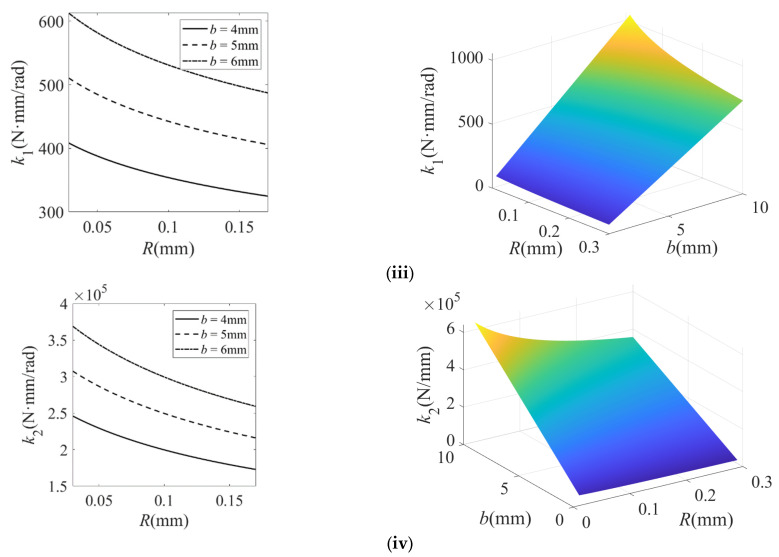
Stiffness characteristics of the filleted straight-beam flexure hinge with respect to radius R, length a, and width b. (**i**) Bending stiffness *k*_1_ varies with a and R; (**ii**) tensile stiffness *k*_2_ varies with a and R; (**iii**) bending stiffness *k*_1_ varies with b and R; (**iv**) tensile stiffness *k*_2_ varies with b and R. In the 3D plot, the color gradient from dark blue, light blue, and light green to light yellow corresponds to the progressive increase in the stiffness of the flexible hinge.

**Figure 7:**
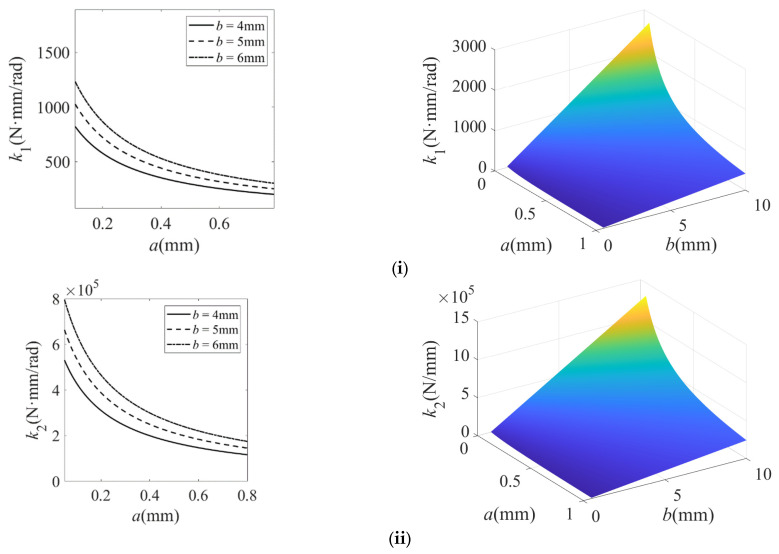
Stiffness characteristics of the filleted straight-beam flexure hinge with respect to length a and width b. (**i**) Bending stiffness *k*_1_; (**ii**) tensile stiffness *k*_2_. In the 3D plot, the color gradient from dark blue, light blue, and light green to light yellow corresponds to the progressive increase in the stiffness of the flexible hinge.

The structural parameters of the filleted straight-beam flexure hinge in the micro-displacement amplification mechanism are summarized in Table 1 and Table 2. Based on the theoretical models in Equations (6) and (9), the calculated stiffness variation of the filleted straight-beam flexure hinge with geometric parameters is presented in Figure 5, Figure 6 and Figure 7.

Based on Figure 5, Figure 6 and Figure 7, the findings of this study are described as follows:

(1) For the filleted straight-beam flexure hinge, both the bending stiffness *k*_1_ and tensile stiffness *k*_2_ increase with the minimum thickness t, albeit with distinct trends: *k*_1_ increases nonlinear growth, while *k*_2_ increases approximately linearly. Furthermore, both *k*_1_ and *k*_2_ are positively correlated with the width b and negatively correlated with the fillet radius R and the straight-beam length a. Among these influencing factors, the minimum thickness t has the most significant impact on *k*_1_ and *k*_2_. The influence of the fillet radius R is relatively small, while the straight-beam length a and width b exert a moderate influence.
(2) For the filleted straight-beam flexure hinge, both the bending stiffness *k*_1_ and tensile stiffness *k*_2_ decrease nonlinearly with increasing fillet radius R and decrease slowly with increasing straight-beam length a. Conversely, *k*_1_ and *k*_2_ increase approximately proportionally to the width b. Among the influencing geometric factors, the straight-beam length a has the most significant effect on both *k*_1_ and *k*_2_. The effect of the width b is subsequently significant, while the influence of the fillet radius R is least pronounced.
(3) For the filleted straight-beam flexure hinge, both the bending stiffness *k*_1_ and the tensile stiffness *k*_2_ decrease nonlinearly with increasing straight-beam length a and increase approximately linearly with increasing width b. In contrast, the straight-beam length a has a more significant impact on both *k*_1_ and *k*_2_, while the width b has a relatively small impact.
(4) For the filleted straight-beam flexure hinge, the bending stiffness *k*_1_ is significantly smaller than the tensile stiffness *k*_2_. This indicates that, within micro-displacement amplification mechanisms, the flexure hinges primarily function as rotational joints connecting rigid links, where their compliance (i.e., low bending stiffness) dominates the motion behavior. Therefore, when calculating hinge stiffness in such mechanisms, the influence of tensile stiffness *k*_2_ can be neglected.

In summary, the minimum thickness t and straight-beam length a exhibit the most significant influence on the stiffness of filleted straight-beam flexure hinge. For the micro-displacement amplification mechanism, the stiffness behavior of flexure hinges can be dominantly characterized by their bending stiffness *k*_1_. Consequently, other stiffness components may be neglected in practical modeling.

## 5. Equivalent Stiffness Derivation for Micro-Displacement Amplification Mechanism

**Figure 8:**
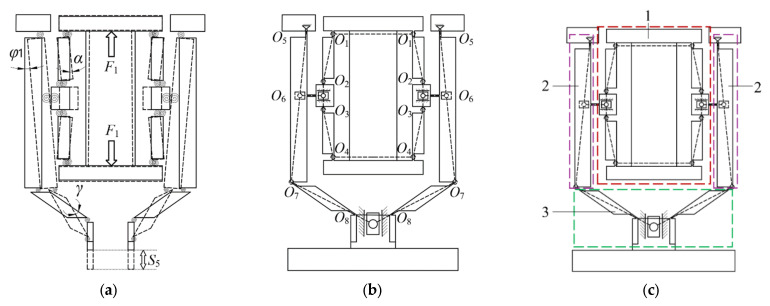
Structure of the micro-displacement amplification mechanism. (**a**) Working process; (**b**) simplified flexible hinge; (**c**) structural components: 1—bridge structure I; 2—lever structure; 3—bridge structure II. Note: The red, purple, and green dashed-line boxes in the figure denote Structure 1, Structure 2, and Structure 3, respectively. The black dashed lines indicate the equivalent lengths of the rigid links, while the black solid lines represent the structural outline of the driving component.

Based on the pseudo-rigid body theory, flexure hinges in micro-displacement amplification mechanism can be modeled as rigid revolute joints. Provided that the material remains within the linear elastic range and deformations follow Hooke’s law, their deformation behavior is characterized by equivalent torsional springs, as shown in Figure 8a.

The micro-displacement amplification mechanism features a symmetrical configuration and consists of 16 filleted straight-beam flexure hinges. During operation, only the deformation of the flexible hinges is considered, while the deformation of the rigid components is ignored. When a rising saw-tooth wave voltage signal is applied to the piezoelectric stack, which elongates along its height direction, an output force F_1_ is generated. This force actuates the amplification mechanism, resulting in a displacement, S_5_, at its base, as illustrated in Figure 8.

**Figure 9:**
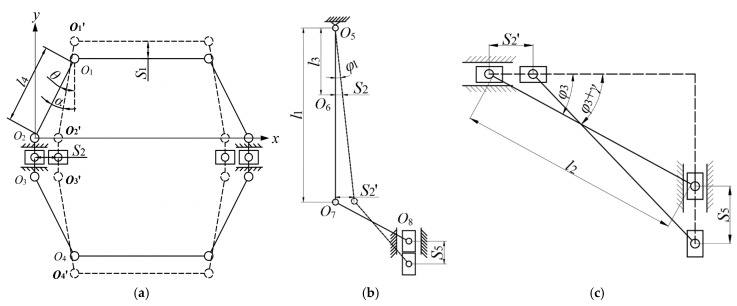
Schematic diagram of each component structure of the micro-displacement amplification mechanism. (**a**) Bridge structure I; (**b**) lever structure; (**c**) bridge structure II.

Figure 9 presents a schematic diagram of the kinematic analysis for the constituent structures of the micro-displacement amplification mechanism.

As shown in Figure 9, when a displacement S_1_ is input from the piezoelectric stack to bridge structure I, this structure undergoes an angular variation α relative to its initial angle *θ*. Given the structural symmetry of bridge structure I, to simplify the analysis, a quarter of its structure is selected for study. Thus:

(10) $$\left\{\begin{array}{l} S_{1}=l_{4}\cos(\theta -\alpha )-l_{4}\cos\theta \\ S_{2}=l_{4}\sin\theta -l_{4}\sin(\theta -\alpha ) \end{array}\right.$$

From Equation (10), the displacement amplification factor C_1_ of the bridge structure I can be obtained as:

(11) $$C_{1}=\frac{S_{2}}{S_{1}}=\frac{l_{4}\sin\theta -l_{4}\sin(\theta -\alpha )}{l_{4}\cos(\theta -\alpha )-l_{4}\cos\theta }$$

Given that the output displacement of the piezoelectric stack is small (only a few micrometers), resulting in a minuscule angular displacement α, the formula can be simplified based on the principle of equivalent infinitesimals:

(12) $$S_{2}=\frac{S_{1}}{\tan\theta }$$

From Figure 9a,b, we know that the output displacement of hinge point O_2_ in the bridge structure I is transferred to hinge point O_6_ of the lever structure. That is, the input displacement of O_6_ is S_2_:

(13) $$\tan\varphi_{1}=\frac{S_{2}}{l_{3}}=\frac{S_{1}}{l_{3}\tan\theta }$$

Although the output displacement S_1_ of the piezoelectric stack is amplified by bridge structure I, which remains limited, the Angle change *φ*_1_ caused by the input displacement S_2_ is very small, so it can be simplified based on the limit theory:

(14) $$\varphi_{1}=\tan\varphi_{1}$$

Thus:

(15) $$\varphi_{1}=\frac{S_{1}}{l_{3}\tan\theta }$$

From the geometric relationship, the output displacement $S_{2}^{\prime }$ at the distal end of the lever structure is given by:

(16) $$S_{2}^{\prime }=\frac{S_{2}l_{1}}{l_{3}}=\frac{S_{1}l_{1}}{l_{3}\tan\theta }$$

As illustrated in Figure 9c, the input displacement to the rigid link O_7_O_8_ in bridge structure II is identical to the output displacement $S_{2}^{\prime }$ at the distal end of the lever structure, which corresponds to the displacement at hinge O_7_. Based on the geometric relationship, the following can be derived:

(17) $$l_{2}\cos\varphi_{3}-l_{2}\cos(\varphi_{3}+\gamma )=S_{2}^{\prime }$$

Substituting Equation (16) into Equation (17) yields:

(18) $$\gamma =\arccos(\cos\varphi_{3}-\frac{S_{1}l_{1}}{l_{2}l_{3}\tan\theta })-\varphi_{3}$$

where *φ*_3_ denotes the initial angle of the rigid link in bridge structure II relative to the horizontal direction, and *γ* stands for the change in the initial angle *φ*_3_.

Under the piezoelectric stack’s output force *F*_1_, the micro-displacement amplification mechanism generates an output displacement S_5_ [41], which is expressed as:

(19) $$S_{5}=l_{1}\cos\frac{180^{\circ }S_{1}}{\pi l_{3}\tan\theta }+\sqrt{{l_{2}}^{2}-e^{2}+2l_{1}e\sin\frac{180^{\circ }S_{1}}{\pi l_{3}\tan\theta }-{l_{1}}^{2}\sin^{2}\frac{180^{\circ }S_{1}}{\pi l_{3}\tan\theta }}-l_{1}-\sqrt{{l_{2}}^{2}-e^{2}}$$

The strain energy stored in each flexure hinge of the micro-displacement amplification mechanism can be expressed as:

(20) $$\left\{\begin{array}{c} A_{\alpha }=\frac{1}{2}k_{1}\alpha^{2}\\ A_{\varphi_{1}}=\frac{1}{2}k_{1}\varphi_{1}^{2}\\ A_{\gamma }=\frac{1}{2}k_{1}\gamma^{2} \end{array}\right.$$

The total strain energy of the mechanism is:

(21) $$E_{d}=8A_{\alpha }+4A_{\varphi_{1}}+4A_{\gamma }$$

The mechanical output work generated by the amplification mechanism driven by piezoelectric force *F*_1_ can be expressed as:

(22) $$W_{F_{1}}=\frac{1}{2}F_{1}S_{5}$$

Combining Equations (21) and (22) based on the energy conservation law yields:

(23) $$F_{1}=\frac{1}{S_{5}}\times (8k_{1}\alpha^{2}+4k_{1}\varphi_{1}^{2}+4k_{1}\gamma^{2})$$

By combining Equations (6), (15), (18), (19) and (23), the equivalent stiffness of the displacement amplification mechanism can be derived as:

(24) $$K=\frac{F_{1}}{S_{5}}=\frac{8k_{1}\alpha^{2}+4k_{1}\varphi_{1}^{2}+4k_{1}\gamma^{2}}{{S_{5}}^{2}}=\frac{Ebt^{3}\left(8\alpha^{2}+n^{2}+p^{2}\right)}{\left(m+12a\right)q^{2}}$$

where:

$$m=\frac{24R\left(\frac{6R^{2}}{t^{2}}+\frac{4R}{t}+1\right)}{{\left(\frac{4R}{t}+1\right)}^{2}\left(\frac{2R}{t}+1\right)}+\frac{144R^{2}\left(\frac{2R}{t}+1\right)\arctan\sqrt{\frac{4R}{t}+1}}{t{\left(\frac{4R}{t}+1\right)}^{\frac{5}{2}}}$$

$$n=\frac{2l_{4}\left(\cos\left(\theta -\alpha \right)-\cos\theta \right)}{l_{3}\tan\theta }$$

$$p=2\left[\arccos\left(\cos\varphi_{3}-\frac{l_{4}l_{1}\left(\cos\left(\theta -\alpha \right)-\cos\theta \right)}{l_{2}l_{3}\tan\theta }\right)-\varphi_{3}\right]$$

$$q=\left[l_{1}\cos\left(\frac{180{}^{\circ}l_{4}\left(\cos\left(\theta -\alpha \right)-\cos\theta \right)}{\pi l_{3}\tan\theta }\right)+\right.\left.g-l_{1}-\sqrt{l_{2}^{2}-e^{2}}\right]$$

$$g=\sqrt{l_{2}^{2}-{\left(l_{1}\sin\left(\frac{180{}^{\circ}l_{4}\left(\cos\left(\theta -\alpha \right)-\cos\theta \right)}{\pi l_{3}\tan\theta }\right)-e\right)}^{2}}$$

## 6. Case Study Analysis of Equivalent Stiffness

**Figure 10:**
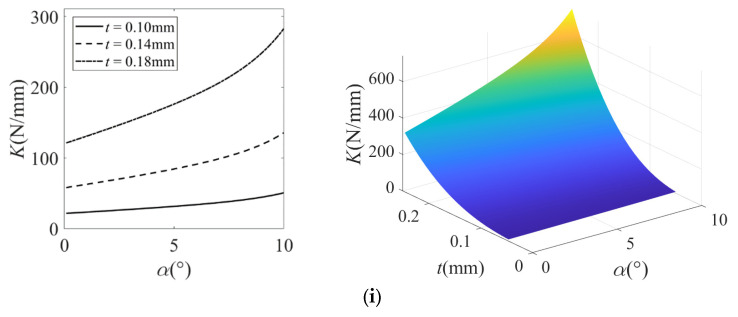

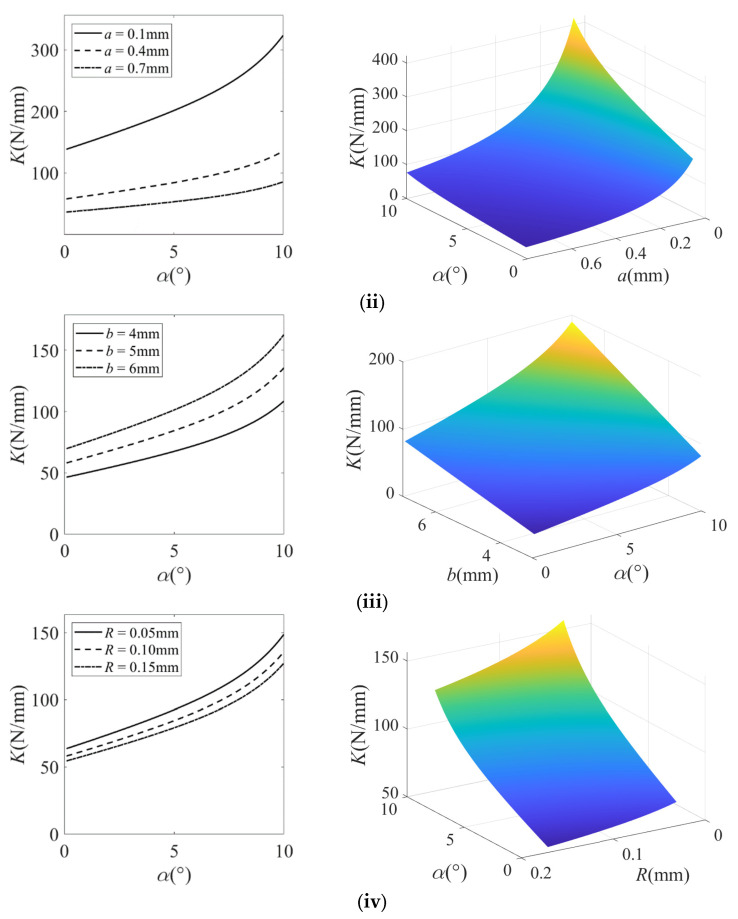
Variation in equivalent stiffness with respect to its key geometric parameters in the micro-displacement amplification mechanism. (**i**) Angular variation α, and thickness t; (**ii**) angular variation α, and length a; (**iii**) angular variation α, and width b; (**iv**) angular variation α, and radius R. In the 3D plot, the color gradient from dark blue, light blue, and light green to light yellow corresponds to the progressive increase in the equivalent stiffness of the micro-displacement amplification mechanism.

The structural parameters of the micro-displacement amplification mechanism and the filleted straight-beam flexure hinge are listed in Table 1, Table 2 and Table 3. Using MATLAB (MATLAB R2021b) analytical methods, the variation in the equivalent stiffness of the micro-displacement amplification mechanism with respect to its key geometric parameters was investigated, with results presented in Figure 10.

From Figure 10, it is determined that:

(1) The equivalent stiffness of the micro-displacement amplification mechanism shows a nonlinear increasing trend with the growing angular variation α of the bridge structure I, and the increase gradually becomes significant. This phenomenon originates from the geometric relationship whereby, when the initial angle *θ* (between the bridge arm and longitudinal axis) and output displacement S_2_ remain constant, the residual angle *θ*-α of bridge structure I decreases continuously with increasing α. Consequently, the component force *F*_1_cos(*θ*-α) of driving force *F*_1_ along the tensile direction of flexible hinges increases significantly, leading to nonlinear enhancement of the mechanism’s equivalent stiffness.
(2) The equivalent stiffness *K* of the micro-displacement amplification mechanism increases with the increasing minimum thickness t and width b of the flexible hinge but decreases as the straight-beam length a and the fillet radius R increase. Among these parameters, the thickness t of the flexible hinge and the straight-beam length a have the most significant influence on the equivalent stiffness, followed by the width b, while the fillet radius R has the least impact. When the width b of the flexible hinge is 5 mm, the fillet radius R is 0.1 mm, the straight-beam length a is 0.4 mm, and the thickness t takes values of 0.1 mm, 0.14 mm, and 0.18 mm respectively, the corresponding equivalent stiffness values are 21.60 N/mm, 57.76 N/mm, and 120.45 N/mm, respectively.

The equivalent stiffness characteristics of the micro-displacement amplification mechanism are closely related to the structural parameters of the flexible hinge. Research shows that the hinge thickness t and straight-beam length a exhibit the most significant effect on the equivalent stiffness. Therefore, to maximize the output displacement of the mechanism for effectively driving the piston motion and minimize the enclosed cavity volume formed by the piston, piston sleeve, and upper bearing seat, thereby generating higher compressed gas pressure to drive rotor rotation, the optimal design strategy should prioritize a smaller hinge thickness t and a larger straight-beam length a.

## 7. Stress Analysis and Experimental Test

### 7.1. Stress Analysis

**Figure 11:**
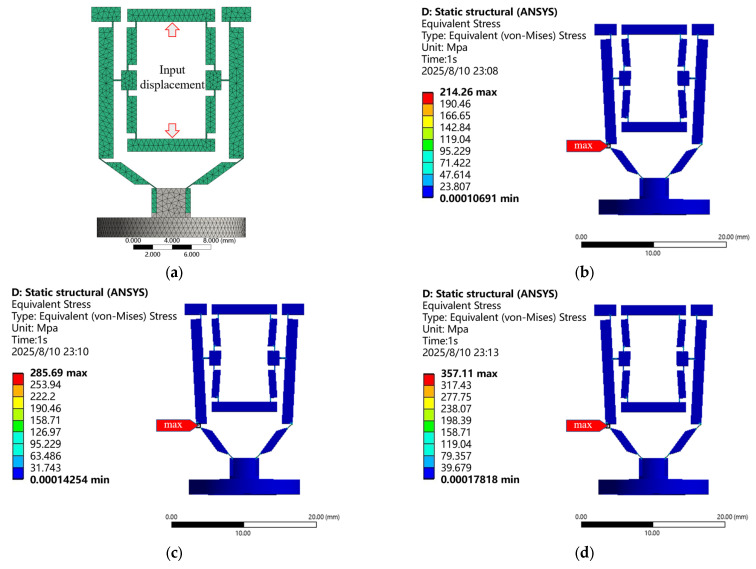
Stress cloud diagrams of the micro-displacement amplification mechanism under varying excitation displacements applied to the piezoelectric actuator. (**a**) Finite element model; (**b**) excitation displacement S_1_ = 0.003 mm; (**c**) excitation displacement S_1_ = 0.004 mm; (**d**) excitation displacement S_1_ = 0.005 mm.

A three-dimensional model of the non-contact piezoelectric actuator micro-displacement amplification mechanism was developed based on its dimensional parameters and imported into ANSYS Workbench 2021R2. Material properties were assigned, meshing was performed, and fixed constraints along with varying excitation displacements were applied. The stress distribution of the mechanism was then calculated through finite element analysis. The simulation results are shown in Figure 11.

Figure 11 shows the von Mises stress distribution in the micro-displacement amplification mechanism of the non-contact piezoelectric actuator under piezoelectric excitations of 0.003 mm, 0.004 mm, and 0.005 mm. The results show that:

(1) The stress of rigid links can be ignored (<39.679 MPa), with stress concentration localized at flexure hinges.
(2) The maximum equivalent stress occurs at the hinge connecting the lever structure and bridge structure II.
(3) When the excitation displacement increased from 0.003 mm to 0.005 mm, the maximum equivalent stress linearly increased from 214.26 MPa to 357.11 MPa, with an increase of 66.7%.

Under the excitation displacement of 0.005 mm, the maximum stress is 357.11 MPa, which is significantly lower than the lower yield strength limit of quenched–tempered 45 steel (500 MPa). The safety factor is calculated as SF = 1.40 (500 MPa/357.11 MPa). Finite element analysis confirms that all hinges remain in the elastic deformation range, with no residual plastic deformation observed after unloading. The stress distribution characteristics and sufficient strength margin fully validate the rationality and reliability of the structural parameter design.

### 7.2. Experimental Test

**Figure 12:**
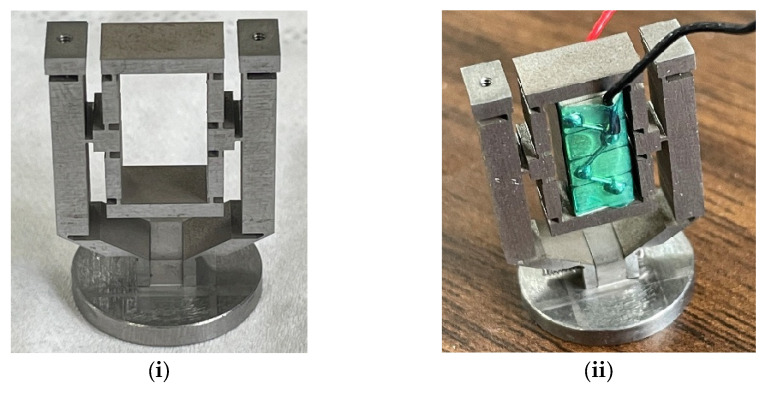
Experimental prototype of the micro-displacement amplification mechanism integrated with the piston. (**i**) Assembly drawing of the amplification mechanism and piston; (**ii**) assembly drawing of the piezoelectric drive system.

The micro-displacement amplification mechanism was fabricated via Wire Electrical Discharge Machining (WEDM), with its output end rigidly connected to the piston. A piezoelectric stack was installed at the mechanism’s input through an interference fit, with the assembled prototype shown in Figure 12.

**Figure 13:**
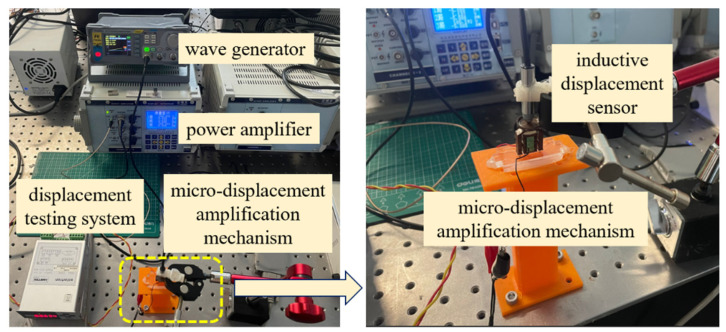
Prototype test of the micro-displacement amplification mechanism.

The operating force and corresponding drive voltage of the piezoelectric stack were selected per the manufacturer’s datasheet specifications. A sawtooth wave signal (frequency: 1 Hz, voltage range: 100–150 V) generated by a waveform generator was amplified and applied to the piezoelectric stack. This induced precise input displacements (0.003 mm, 0.004 mm, 0.005 mm) along the height direction at the amplification mechanism’s input. The amplified displacement drove piston motion, while a high-precision inductive displacement sensor measured the piston’s output displacement. The equivalent stiffness of the amplification mechanism was then derived from these measurements. The test configuration is illustrated in Figure 13, with experimental results and theory–experiment error comparisons detailed in Table 4 and Table 5.

Table 4 and Table 5 demonstrate that:

(1) The output displacement of the micro-displacement amplification mechanism increases with the input displacement, while the equivalent stiffness remains relatively constant. When the input displacement increases from 0.003 mm to 0.005 mm, the output displacement rises from 0.0694 mm to 0.1251 mm. Meanwhile, the equivalent stiffness test value exhibits minimal variation, decreasing slightly from 132.72 N/mm to 131.43 N/mm.
(2) The measured equivalent stiffness values are consistently higher than the theoretical values, indicating a certain degree of deviation. Furthermore, the magnitude of this deviation decreases as the input displacement increases. Specifically, when the input displacement increases from 0.003 mm to 0.005 mm, the error reduces from 19.03% to 18.23%, representing a decrease of 4.2%.
(3) The experimental average equivalent stiffness of the micro-displacement amplification mechanism is 132.04 N/mm compared to the theoretical value of 107.464 N/mm, yielding an average error of 18.61%.

The discrepancy between experimental and theoretical equivalent stiffness values in the micro-displacement amplification mechanism may originate from the following factors:

(1) Machining accuracy factors. Machining errors in components such as the flexible hinges at the joints of the rigid links and the interference fit interfaces with the piezoelectric stack within the micro-displacement amplification mechanism lead to a partial counteraction of the displacement generated by the piezoelectric stack. This reduces the effective displacement output amplified and transmitted by the mechanism, ultimately resulting in a higher equivalent stiffness value calculated based on experimental data.
(2) Experimental error factors. Factors such as the fixture method for both the micro-displacement amplification mechanism and the displacement sensor, the inherent accuracy of the displacement sensor, and the coupling effect of vibrations from various test instruments on the workbench during experimentation can all affect the measurement accuracy of the mechanism under small-displacement conditions. Therefore, utilizing these experimentally compromised measurements to calculate the equivalent stiffness of the micro-displacement amplification mechanism will lead to an overestimation of the stiffness value.

To address these error factors, the following mitigation measures will be implemented:

(1) Manufacturing process enhancement. Higher-precision machining techniques will be employed to fabricate the micro-displacement amplification mechanism, thereby reducing geometric accuracy deviations (such as dimensional error, form error, positional error, etc.) in its rigid links, flexible hinges and piezoelectric stack contact interfaces.
(2) Experimental testing enhancement. A dedicated fixture will be developed to rigidly mount the micro-displacement amplification mechanism, enabling precise measurement of its output force when the piezoelectric stack is driven. Instrumentation fixtures with integrated vibration-damping capability will be fabricated to mitigate environmental interference during testing. High-precision contact-type force sensors and non-contact displacement sensors will be employed to reduce measurement errors and enhance the accuracy of equivalent stiffness characterization for the mechanism.

## 8. Conclusions

In this study, a novel non-contact piezoelectric actuator is proposed, its working principle is clarified, and a mathematical model of the micro-displacement amplification mechanism employing filleted straight-beam flexure hinges is established. The analytical equations for the bending stiffness and tensile stiffness of the flexure hinge, along with the equivalent stiffness of the amplification mechanism, are derived. Using these equations, the influence of flexure hinge geometric parameters on these stiffness characteristics is systematically investigated. Finite element analysis of the stress distribution in the micro-displacement amplification mechanism verifies the rationality and reliability of its structural design. The prototype has been fabricated, and its equivalent stiffness tests are conducted. Results show:

(1) Increasing the minimum thickness and width of the flexure hinges significantly enhance stiffness, whereas extending the straight-beam length or fillet radius reduces stiffness. Among these parameters, minimum thickness and straight-beam length have the most significant influence on the stiffness.
(2) The tensile stiffness of the flexure hinges is significantly larger than their bending stiffness. Therefore, when calculating the equivalent stiffness of the micro-displacement amplification mechanism with filleted straight-beam flexure hinges, the bending stiffness may be used as an approximation for the overall hinge stiffness.
(3) The thickness and width of the flexure hinges exhibit significantly stronger effects on the equivalent stiffness of the micro-displacement amplification mechanism, whereas the fillet radius and straight-beam length demonstrate relatively less pronounced influence.
(4) The maximum equivalent stress in the flexure hinges of the micro-displacement amplification mechanism (particularly at the lever-bridge II junction) increases with excitation displacement. This remains significantly below the yield strength of the selected material, with a safety factor reaching 1.40. Finite element analysis confirms that all hinges operate within the elastic deformation range, exhibiting no residual plastic deformation after unloading. Stress in rigid links is negligible, thus validating their structural safety and reliability.
(5) The experimental value of the equivalent stiffness of the micro-displacement amplification mechanism is 132.04 N/mm, which is 18.61% higher than the theoretical value of 107.464 N/mm. Based on a systematic error attribution analysis, optimization and improvement strategies have been proposed. The theoretical calculation is basically consistent with the experimental results, confirming the accuracy of the stiffness model.

## Figures and Tables

**Table 1:** Geometric parameters of the filleted straight-beam flexure hinge.

a (mm)	b (mm)	t (mm)	R (mm)
0.4	5	0.14	0.1

**Table 2:** Material parameters of the filleted straight-beam flexure hinge.

Density (kg/m^3^)	Young’s Modulus (MPa)	Tensile Strength (MPa)	Poisson’s Ratio
7.85 × 10^3^	2 × 10^5^	4 × 10^2^	0.3

**Table 3:** Geometric parameters of the micro-displacement amplification mechanism.

*θ* (°)	*e*^1^ (mm)	*l*_1_ (mm)	*l*_2_ (mm)	*l*_3_ (mm)	*l*_4_ (mm)	*φ*_3_ (°)
8	6	15	7	5	5.05	31

^1^ e denotes the bridge structure II’s rigid links horizontal projection length.

**Table 4:** Comparison of theoretical and experimental results of the micro-displacement amplification mechanism.

Input Value (mm)	Output Value (mm)	Experimental Equivalent Stiffness (N/mm)	Theoretical Equivalent Stiffness (N/mm)	Error (%)
0.003	0.0694	132.72	107.464	19.03
0.004	0.0959	131.96	107.464	18.56
0.005	0.1251	131.43	107.464	18.23

**Table 5:** Comparison of theoretical and experimental mean values for the equivalent stiffness of the micro-displacement amplification mechanism.

Theoretical Value (N/mm)	Experimental Mean Value (N/mm)	Error (%)
107.464	132.04	18.61%

## Data Availability

All relevant data are included in the article.
